# Mimicking exposures to acute and lifetime concentrations of inhaled silver nanoparticles by two different in vitro approaches

**DOI:** 10.3762/bjnano.5.149

**Published:** 2014-08-26

**Authors:** Fabian Herzog, Kateryna Loza, Sandor Balog, Martin J D Clift, Matthias Epple, Peter Gehr, Alke Petri-Fink, Barbara Rothen-Rutishauser

**Affiliations:** 1 Adolphe Merkle Institute, BioNanomaterials, University of Fribourg, Rte de l'Ancienne Papeterie, CP 209, 1723 Marly, Switzerland; 2 Inorganic Chemistry and Center for Nanointegration Duisburg-Essen (CeNIDE), University of Duisburg-Essen, Universitaetsstrasse 5–7, 45141 Essen, Germany; 3 Institute of Anatomy, University of Bern, Baltzerstrasse 2, 3012 Bern, Switzerland; 4 Department of Chemistry, University of Fribourg, Chemin du Musée 9, 1700 Fribourg, Switzerland; 5 Respiratory Medicine, Department of Clinical Research, Inselspital University Hospital, University of Bern, Murtenstrasse 50, 3008 Bern, Switzerland

**Keywords:** air–liquid exposure, dosimetry, lung cells in vitro, silver nanoparticles, toxicity

## Abstract

In the emerging market of nano-sized products, silver nanoparticles (Ag NPs) are widely used due to their antimicrobial properties. Human interaction with Ag NPs can occur through the lung, skin, gastrointestinal tract, and bloodstream. However, the inhalation of Ag NP aerosols is a primary concern. To study the possible effects of inhaled Ag NPs, an in vitro triple cell co-culture model of the human alveolar/airway barrier (A549 epithelial cells, human peripheral blood monocyte derived dendritic and macrophage cells) together with an air–liquid interface cell exposure (ALICE) system was used in order to reflect a real-life exposure scenario. Cells were exposed at the air–liquid interface (ALI) to 0.03, 0.3, and 3 µg Ag/cm^2^ of Ag NPs (diameter 100 nm; coated with polyvinylpyrrolidone: PVP). Ag NPs were found to be highly aggregated within ALI exposed cells with no impairment of cell morphology. Furthermore, a significant increase in release of cytotoxic (LDH), oxidative stress (SOD-1, HMOX-1) or pro-inflammatory markers (TNF-α, IL-8) was absent. As a comparison, cells were exposed to Ag NPs in submerged conditions to 10, 20, and 30 µg Ag/mL. The deposited dose per surface area was estimated by using a dosimetry model (ISDD) to directly compare submerged vs ALI exposure concentrations after 4 and 24 h. Unlike ALI exposures, the two highest concentrations under submerged conditions promoted a cytotoxic and pro-inflammatory response after 24 h. Interestingly, when cell cultures were co-incubated with lipopolysaccharide (LPS), no synergistic inflammatory effects were observed. By using two different exposure scenarios it has been shown that the ALI as well as the suspension conditions for the lower concentrations after 4 h, reflecting real-life concentrations of an acute 24 h exposure, did not induce any adverse effects in a complex 3D model mimicking the human alveolar/airway barrier. However, the highest concentrations used in the ALI setup, as well as all concentrations under submerged conditions after 24 h, reflecting more of a chronic lifetime exposure concentration, showed cytotoxic as well as pro-inflammatory effects. In conclusion, more studies need to address long-term and chronic Ag NP exposure effects.

## Introduction

Silver possesses antiseptic and germicidal properties [1]. These effects are enhanced in combination with the possibilities of nanotechnology, when silver is manufactured as particles at the nanoscale. Defined as objects with all three external dimensions between 1 and 100 nm [2], silver nanoparticles (Ag NPs) allow for a vast range of applications and are the most commonly used material in the emerging markets of nano-sized products [3–6]. Consumer applications using Ag NPs as antimicrobial agents vary from incorporations into materials such as for textile fabrics, medical devices, air filters, and food containers, to dispersions, e.g., for water disinfectants and cosmetics (e.g., deodorants), as well as many more commodities for ‘everyday use’ [7–8].

The application of Ag NPs leading to their mass use and production contrasts with the lack of profound knowledge regarding their biological interaction, including possible adverse health effects of Ag NPs and NPs in general [9–10]. An essential biological role of Ag has not been reported so far, but toxic effects to a number of organisms have been demonstrated [11]. Therefore, the effects of Ag NPs on human health and the environment are currently increasingly explored [12]. Human interaction with Ag NPs can occur through the lung, skin, gastrointestinal tract, and bloodstream. However, inhalation of Ag NPs is a primary concern for humans in an occupational environment [13]. Inhalation, or ingestion, of Ag in large quantities and over a long period of time can cause a disease called “argyria”, which leads to a blue or grey discoloration of the skin and other organs [14]. However, many questions remain open concerning the specific interactions of Ag NPs with organisms at the biochemical and cellular level [15]. For example, it is still unclear whether the effects of Ag are a direct result of the NPs themselves or should rather be attributed to the interaction with Ag ions (Ag^+^) [16–17] that are released from the NPs [18]. In aqueous environments, metallic Ag NPs oxidize, thereby releasing Ag^+^. Ag oxidation is a slow process that strongly depends on the properties of the Ag NPs (such as size, surface and coating of the NPs) and on the environmental conditions [18]. Several studies have already reported upon this ambiguous question, such as [1,11,15].

It has been reported that cytotoxicity and (pro)-inflammatory cytokine release could be observed upon in vitro exposures of Ag NPs to a variety of cell types including immune cells (such as macrophages and monocytes [19–21]) and epithelial lung cells [22–24]. Furthermore, increased levels of oxidative stress and reactive oxygen species (ROS) were detected over a time period of 48 h [22,25–26]. Environmental stressors trigger the production of intracellular ROS, which can overwhelm the cellular antioxidant defence system. ROS can cause DNA damage, which results in the breaking of DNA strands and covalent DNA modifications [10]. Hence, Ag NPs have been shown to cause significant DNA damage in human lung cells in vitro [25,27] suggesting a potential genotoxic mechanism. Despite this, the specific interaction of Ag NPs with cells still remains unclear.

Ag NPs released into the environment (such as for instance air or water, experimental media or biological fluids) are subject to a number of processes (such as aggregation and oxidation with the formation of Ag^+^) that alter their physico-chemical characteristics. These possible processes influence the mode of transport, fate and possible toxicity of Ag [28]. Recent studies highlight the contradicting elements that may contribute to the biological impact of Ag NPs such as shape and size [29–30], surface chemistry [31–32] or a combined mechanism of particle and ions [33]. As the literature is contradicting and the biological interaction of Ag exposures is still unknown, it is essential to perform in-depth research to estimate the potential properties of Ag NPs for safe commercial applications.

To investigate Ag NP–lung interactions, different experimental approaches are used. In animal models, NPs can be applied via instillation [34] or by inhalation [35]. In order to reduce the number of animals used for research, continuous efforts are made towards sophisticated in vitro methods for toxicology testing [36]. The most commonly used in vitro setup for many studies are submerged cultured lung cells [30,36–38]. However, they do not reflect real-life conditions in the lung when Ag NPs are inhaled as an aerosol. Therefore, our group has developed and evaluated a sophisticated 3D model of the human epithelial alveolar/airway barrier in vitro, which is composed of epithelial cells, human monocyte-derived macrophages (MDMs) and dendritic cells (MDDCs) [39]. The model is reflecting a realistic cellular scenario in the lung, as it is designed for direct exposure of cells to an aerosol [40]. Together with a dose-controlled air–liquid interface cell exposure (ALICE) system [41] the possible adverse effects of zinc oxide [41], gold (Au) [42–43] and Ag NPs as well as Ag ions [44] have been evaluated. The aim of the present study was therefore to use a recently established system [44] to assess the effects of Ag NPs and Ag^+^ on the 3D lung model. Compared to the citrate-coated Ag NPs in the previous study [44] we used polyvinylpyrrolidone (PVP)-coated Ag NPs with a larger size. Those particles are well characterized and have been previously used in other studies [45–46]. In addition, the results between air–liquid interface (ALI) and submerged exposures to different concentrations were performed in order to reveal a greater insight into the effect of Ag NP toxicity.

## Results

### Particle characterisation

**Figure 1:**
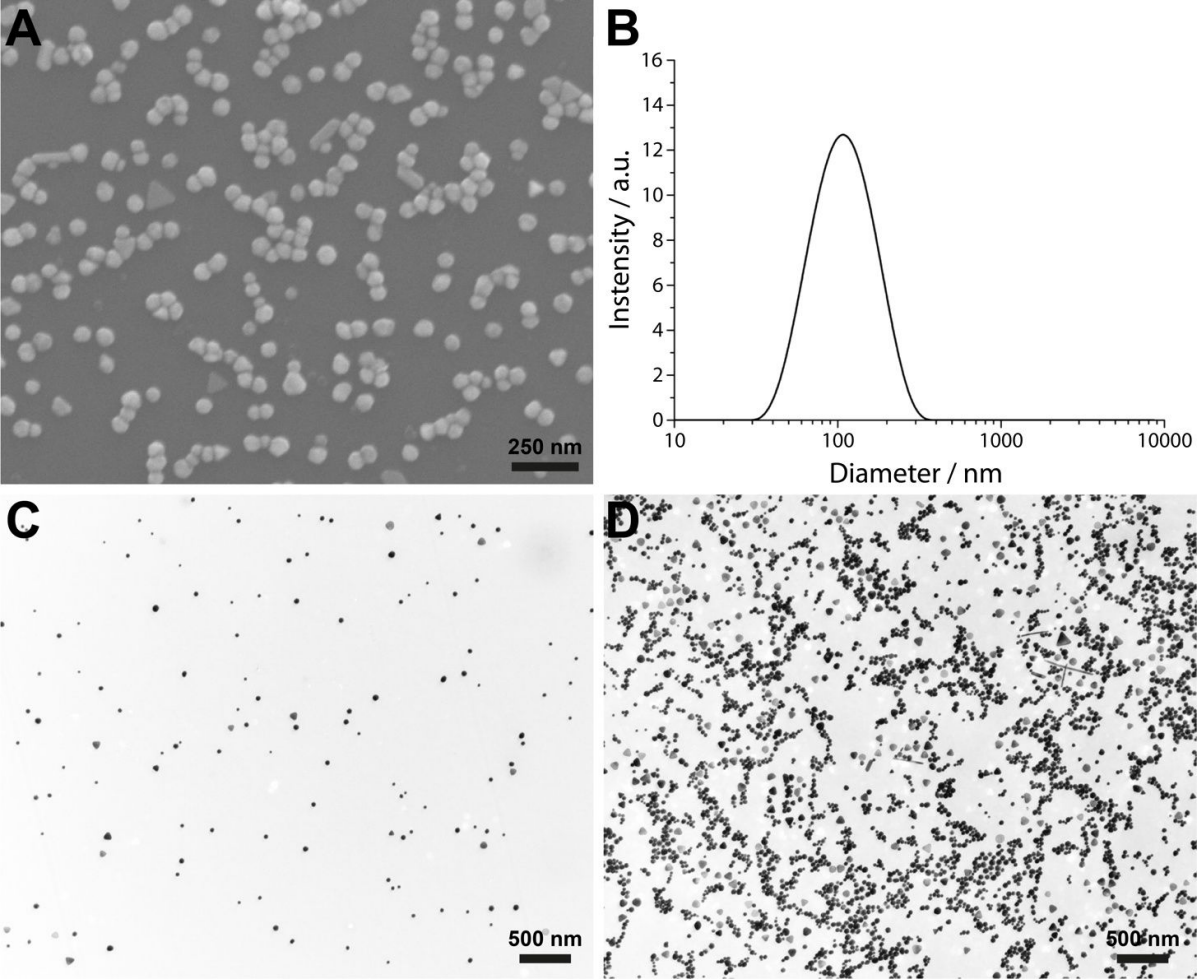
Scanning electron microscopic image (A) of Ag NPs deposited on a silicon wafer. The particle size distribution (B) was measured by dynamic light scattering and showed an average hydrodynamic diameter of 116 ± 7 nm and a zeta potential of −20 ± 5 mV. Ag NPs deposited at a concentration of 0.03 µg Ag/cm^2^ (C) and 3 µg Ag/cm^2^ (D) after aerosolisation on transmission electron microscopy grids.

PVP-coated Ag NPs were characterised by scanning electron microscopy (SEM) and dynamic light scattering (DLS). Figure 1A shows a representative SEM image of particles deposited on a silicon wafer and Figure 1B the particle size distribution as measured with DLS.

In order to compare this study with previous works using Au and Ag NPs (20 nm) with the same setup [42,44] the stock solutions (1.5 mg Ag/mL) were diluted to 24 and 240 μg Ag/mL, respectively, and concentrated by ultrafiltration to 2.4 mg Ag/mL. The Ag NP suspension (1 mL) was nebulized in the ALICE system. The amount of deposited Ag was calculated by the efficiency of the system (50% as previously calculated for smaller Ag NPs [44]) and the quartz crystal microbalance giving 0.03, 0.3, and 3 µg Ag/cm^2^, respectively. Exposures of Ag NPs onto transmission electron microscopy (TEM) grids (Figure 1C and 1D) were used to visualize the uniformity of the depositions and to detect whether Ag NPs agglomerate by the nebulization process. Ag NPs were found not to agglomerate solely by aerosolisation and to be deposited homogeneously.

### Ag NP exposure and dose determination

Triple cell co-cultures composed of A549 cells, MDMs, and MDDCs [40,42] were exposed at the ALI, similarly as described in [44], to three different concentrations of Ag NPs with a final areal density of 0.03, 0.3, and 3 µg Ag/cm^2^. Furthermore, cells were exposed under submerged conditions with concentrations of 10, 20, and 30 µg Ag/mL. In case of NPs, the motion of which is governed by different kinetics than that of soluble components, the in vitro dose delivered to the cells is generally not equal to the administered dose. To account for the kinetics of NPs, the amount of Ag NPs delivered to the cell over time was estimated with a recently developed in vitro sedimentation, diffusion, and dosimetry (ISDD) model [47] and was compared with the result of the ALI exposures.

According to the ISDD model, for submerged exposures over 4 h with Ag NP concentrations of 10, 20, and 30 µg Ag/mL, at least 24% of the incubated NP fraction is expected to be delivered to the cell layer, resulting in surface concentrations of 0.6, 1.1, and 1.7 µg Ag/cm^2^, respectively. After 24 h of incubation, at least 71% of the exposed NP fraction is expected to be delivered, resulting in increased surface concentrations of 1.7, 3.4, and 5.1 µg Ag/cm^2^. Therefore, the two exposure scenarios could be compared due to similar mass deposition on the lung cells surface.

### Cell morphology and particle uptake

**Figure 2:**
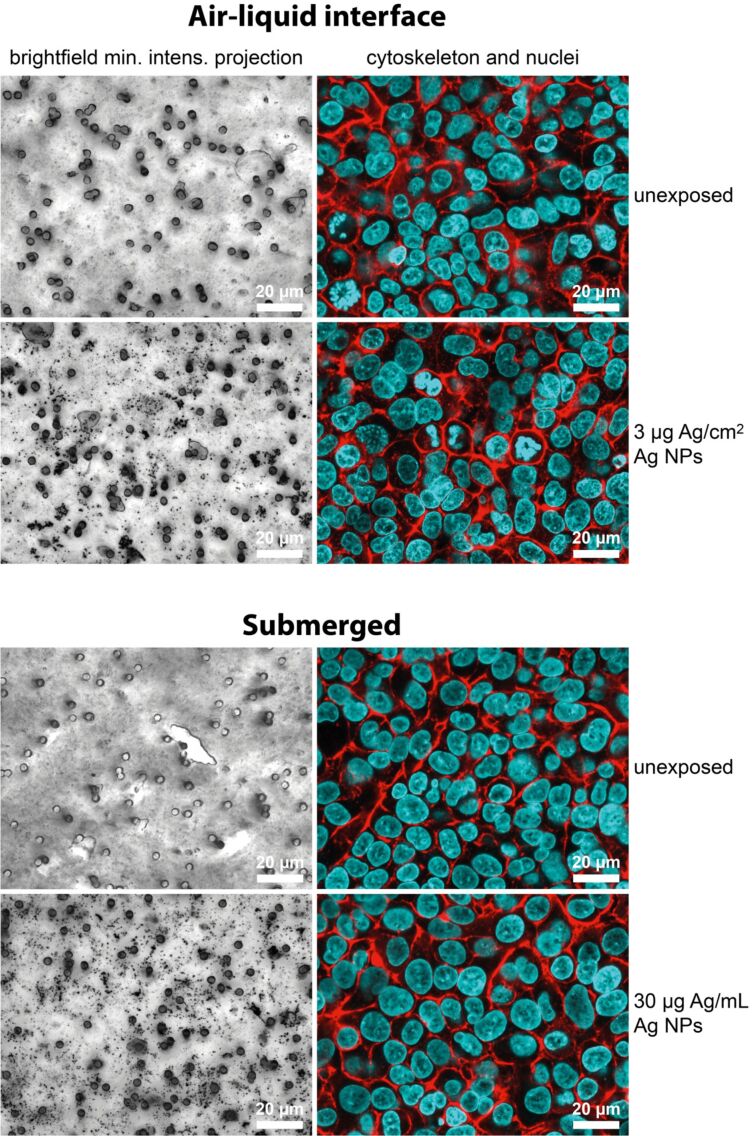
Illustrated are triple cell co-cultures at the air–liquid interface and under submerged conditions, unexposed and exposed to 3 µg Ag/cm^2^ or 30 µg Ag/mL Ag NPs, respectively. At 24 h post-exposure time, the cells were fixed and stained for actin (phalloidin rhodamine; red) and DNA (DAPI; blue). xy-Stack minimum intensity projections of phase contrast images are shown on the left side and fluorescence xy projections of single optical slices on the right.

The cell morphology was studied with laser scanning microscopy (LSM) (Figure 2). The exposure to Ag NPs (i.e., either ALI or submerged) did not alter the morphology shown by staining F-actin with phalloidin rhodamine (red), nor could any DNA alterations be observed as visualized with DAPI (blue). Minimum intensity projections of z-stack phase contrast images revealed NPs either inside the cells or attached to the cell surface. Due to the cultivation of the cells on cell culture inserts, the pores of the insert membranes become visible. Compared to unexposed cells, large NP aggregates can be seen as dark spots in Ag NP-treated cell cultures.

**Figure 3:**
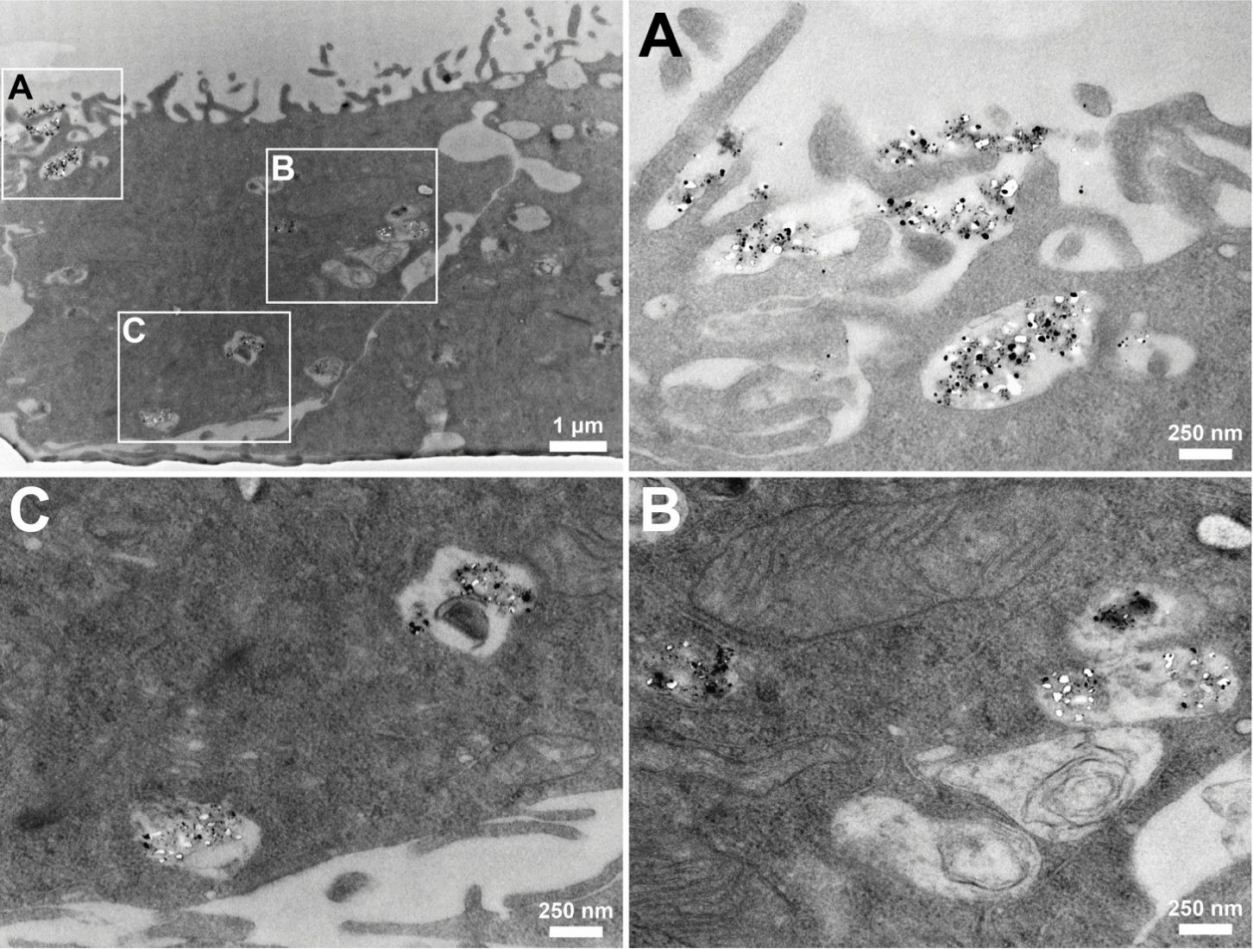
Ag NP aggregates were found in the upper cell layer of the transwell membrane. A representative image of the cell layer 24 h after submerged exposure to 20 µg Ag/mL Ag NPs is shown at low magnification (top left). Higher magnifications of the black marked boxes (A–C) revealed Ag NP aggregates attached to cells (A) and in vesicles of cells (B, C).

TEM was used to resolve the Ag NPs taken up by cells and to determine the shape and agglomeration state of the NPs. The cells were exposed in submerged conditions to 20 µg Ag/mL Ag NPs and fixed 24 h after exposure for TEM. In Figure 3 large aggregates attached to cells and within vesicles are visible in those cells present in the upper layer of the triple cell co-culture model, which is a similar pattern as we have observed for Ag NPs exposed to cells at the ALI [44]. To reduce misinterpretation due to staining artefacts [48], samples were treated with uranyl acetate only, without lead citrate.

### Cytotoxicity

**Figure 4:**
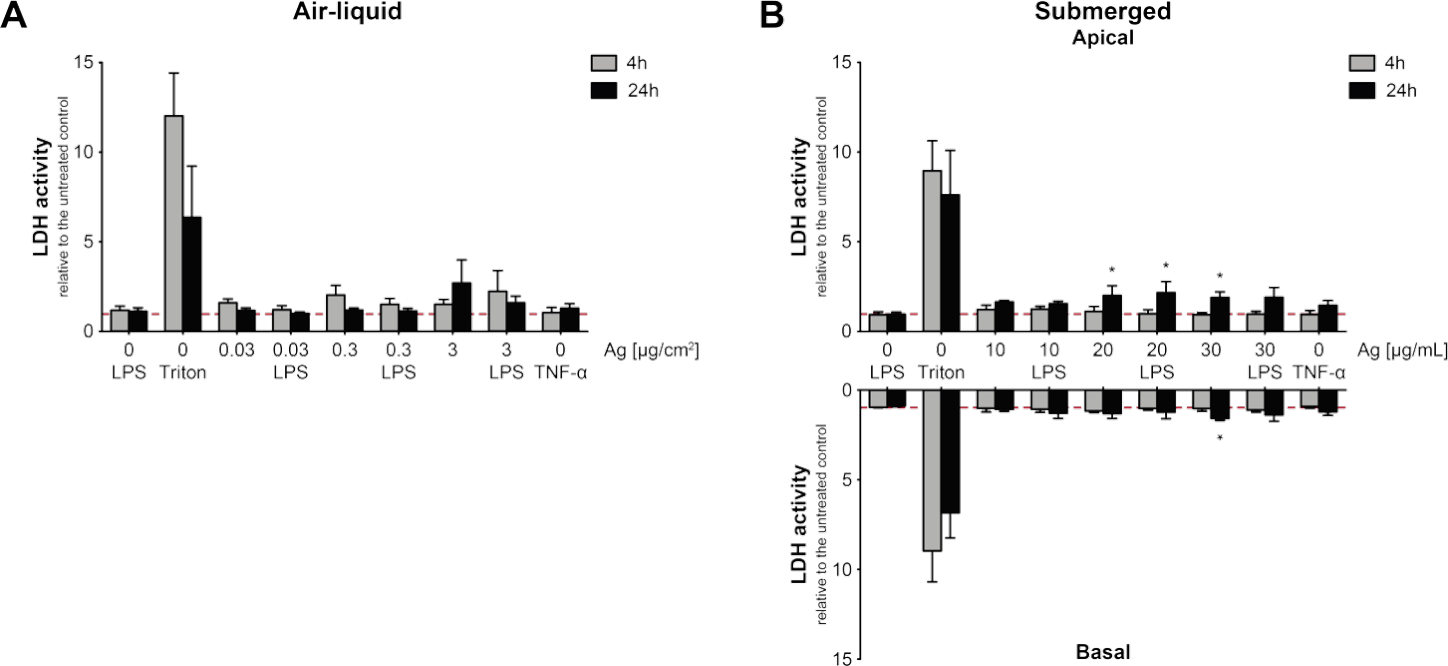
Extracellular LDH release was quantified relative to the untreated control (reference: red dashed line = 1.0) 4 h (grey bars) and 24 h (black bars), respectively, after exposure. Air–liquid interface (A) and submerged exposures (B) were analysed. Supernatants of submerged cultures in the lower (basal) and the upper (apical) transwell were individually evaluated. Incubation with Triton X-100 for 4 and 24 h were used as positive control (Triton). Controls for ELISA (TNF-α and LPS) were additionally analysed to exclude false positive results. Error bars represent the standard error of the mean (SEM) for at least four independent experiments. A two-way analysis of variance (ANOVA) with a subsequent Dunnett’s post-hoc test was performed. Values were considered significantly different with *p* < 0.05 (*). For detailed statistical information see Supporting Information File 1.

As described in [44], we measured the release of lactate dehydrogenase (LDH) as a marker for the cell integrity to assess the cytotoxic potential of cells exposed to Ag NPs. Also included were cultures incubated with lipopolysaccharide (LPS) as a pro-inflammatory stimulus and tumor necrosis factor alpha (TNF-α) as positive control for interleukin-8 (IL-8) release to exclude false positive results. Cells were lysed with Triton X-100 as positive control. All values are relative to unexposed negative controls and the reference point (1.0) is indicated as red dashed line (Figure 4). Statistical information can be found in Supporting Information File 1 (Figure S1 and Figure S2).

Cells exposed at the ALI did not show a significant LDH release for all three deposited Ag NP concentrations of 0.03, 0.3, and 3 µg Ag/cm^2^ 4 and 24 h after exposure (Figure 4A). However, elevated levels of LDH were observed at 3 µg Ag/cm^2^ after 24 h with 2.7 ± 1.3 fold (*p* = 0.056) relative to the negative control. LDH levels for cells exposed under submerged conditions were determined in the upper compartment of the transwell insert as well as in the lower compartment (Figure 4B). Due to differences in the amount of cell culture medium used in the different compartments the values were adjusted according to the appropriate dilution factor. A concentration-dependent release of LDH could only be detected after 24 h. Ag NP exposures of 20 and 30 µg Ag/mL significantly increased the relative LDH activity in the upper compartment to 2.0 ± 0.5 fold (*p* = 0.024) and 1.9 ± 0.3 fold (*p* = 0.048), respectively. A significant increase could also be measured for LPS-stimulated and co-exposure with 20 µg Ag/mL Ag NPs indicated by a value of 2.2 ± 0.6 fold (*p* = 0.026). In the lower compartment exposure of 30 µg Ag/mL resulted in an elevated LDH level of 1.6 ± 0.1 fold (*p* = 0.025) relative to the negative control. As an assay control we analysed PBS and Triton X-100 treated positive control samples mixed with Ag NP concentrations of 10, 20 and 30 µg Ag/mL. A concentration-dependent decrease of relative LDH could be observed in positive control solutions (data not shown).

### Cytokine/chemokine secretion

**Figure 5:**
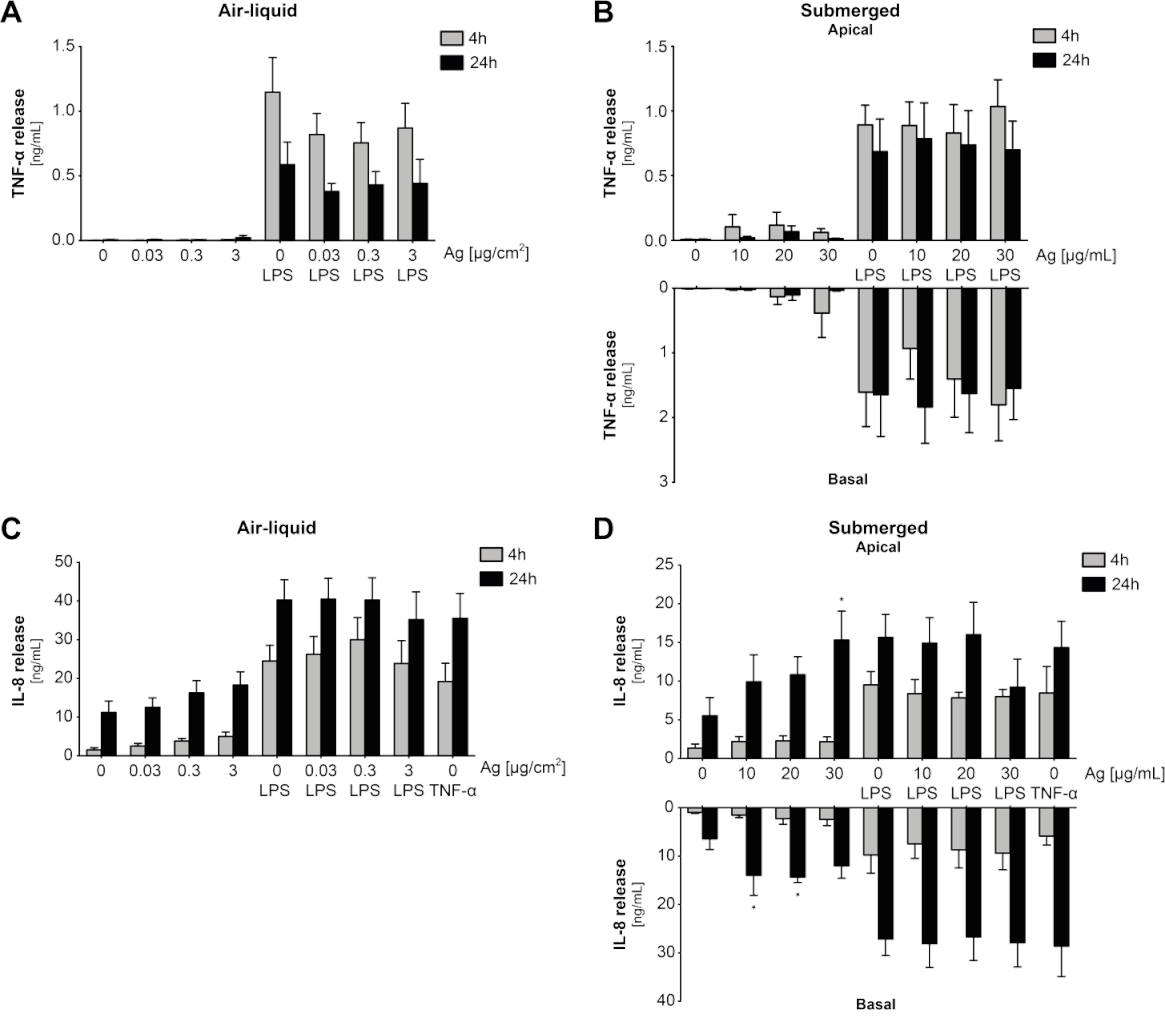
The extracellular release of pro-inflammatory markers was analysed by ELISA. Excreted TNF-α (A and B) and IL-8 (C and D) were quantified (ng/mL) 4 and 24 h after exposure (grey bars and black bars, respectively). Cells were exposed at the air–liquid interface (A and C) and under submerged conditions (B and D). Supernatants of submerged cultures in the lower (basal) and the upper (apical) transwell were individually analysed. Error bars represent the SEM for at least four independent experiments. A two-way ANOVA with a subsequent Dunnett’s post-hoc test was performed. Values were considered significantly different with *p* < 0.05 (*). For detailed statistical information see Supporting Information File 1.

As described in [44], the release of the pro-inflammatory markers TNF-α and IL-8 was measured 4 and 24 h after exposure by enzyme linked immunosorbent assay (ELISA) to characterize the pro-inflammatory response of the cell culture lung model (Figure 5). Unexposed cells served as negative controls. As positive controls, 1 µg/mL LPS was used to trigger an immune response, and 15 ng/mL TNF-α was applied to stimulate IL-8 secretion. Furthermore, LPS-treated cells were co-exposed to Ag NPs to study NP effects under inflammatory conditions as already described [42]. Statistical information can be found in Supporting Information File 1 for TNF-α (Figure S3 and Figure S4) and IL-8 (Figure S5 and Figure S6).

For cells exposed to Ag NPs at the ALI to 0.03, 0.3, and 3 µg Ag/cm^2^ a moderate TNF-α concentration could only be detected 24 h after exposure to 3 µg Ag/cm^2^ (Figure 5A). When unexposed cells were stimulated with LPS the concentration of TNF-α increased after 4 and 24 h, respectively. However, reduced values were measured after 24 h. Exposed cells co-stimulated with LPS did not lead to any synergistic effects and the concentrations were statistically not different from LPS-treated cells only (i.e., no NP exposure). Furthermore, a similar pattern was observed with a maximum after 4 h and a reduced increase after 24 h. Cells exposed to 10, 20, and 30 µg Ag/mL under submerged conditions did not show a statistically significant release of TNF-α under any conditions applied (Figure 5B). When treated with LPS a TNF-α release could be observed similar as for ALI cell cultures, which did not differ from Ag NP–LPS co-exposed cells.

ALI-exposed cells did not show an Ag NP-related release of IL-8 (Figure 5C). However, the amount of secreted IL-8 increased from 4 to 24 h, which could also be detected in negative controls. The amount of IL-8 released for treated cells was statistically not different from unexposed cells. Upon treatment with both LPS and TNF-α secretion of IL-8 was stimulated as measured after 4 and 24 h, respectively. Particle-exposed cells did not show synergistic effects upon immune stimulation with LPS and the release of IL-8 was statistically not different to unexposed cells after 4 and 24 h post-exposure, respectively. After 24 h exposure under submerged conditions an increased release of IL-8 could be detected at the apical side in the upper transwell insert (Figure 5D), which was statistically significant at the highest dose of 30 µg Ag/mL (15 ± 4 ng/mL; *p* = 0.011). Furthermore, at the basal side in the lower compartment a significant increase of IL-8 release could already be measured after 24 h at 10 µg Ag/mL (14 ± 4 ng/mL; *p* = 0.038) and 20 µg Ag/mL (14 ± 1 ng/mL; *p* = 0.028). At 30 µg Ag/mL however, the measured concentration was not significant anymore due to the high standard deviation. Similar as for ALI exposed cells, treatment with LPS and TNF-α stimulated the release of IL-8 but no synergistic effects could be observed when simultaneously exposed with Ag NPs. Ag NPs were not found to interfere with the ELISA assay (data not shown).

### Gene expression of pro-inflammatory and oxidative stress markers

**Figure 6:**
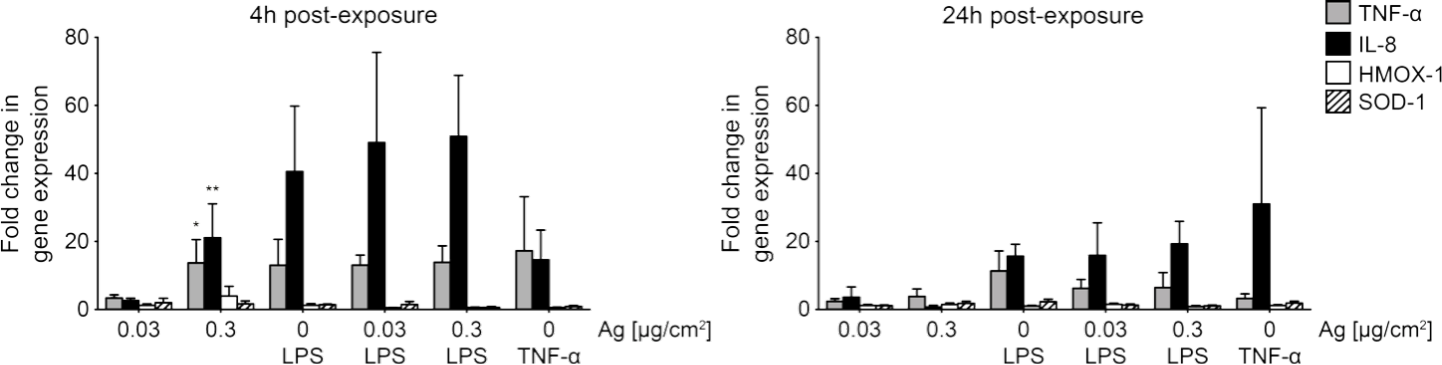
4 and 24 h after cell exposure, the total RNA content was collected. Subsequent analysis by real-time RT-PCR revealed the mRNA induction levels of pro-inflammatory markers TNF-α (grey) and IL-8 (black), as well as oxidative stress markers SOD-1 (white) and HMOX-1 (striated). The transcriptional activities relative to *GAPDH* expression levels of the marker genes are expressed as fold changes (2^−ΔΔCt^). Error bars represent the SEM for at least three independent experiments. A two-way ANOVA with a subsequent Dunnett’s post-hoc test was performed. Values were considered significantly different with *p* < 0.05 (*), *p* < 0.01 (**). For detailed statistical information see Supporting File 1.

As described in [44], the total RNA content of the triple cell co-cultures was collected 4 and 24 h after ALI exposures to analyse a pro-inflammatory and oxidative stress response (Figure 6). The relative mRNA expression for the pro-inflammatory markers TNF-α and IL-8 and the oxidative stress markers superoxide dismutase 1 (SOD-1) and heme oxygenase 1 (HMOX-1) were evaluated. Fold changes of induction relative to glyceraldehyde 3-phosphate dehydrogenase (*GAPDH*) and in comparison to unexposed controls (2^−ΔΔCt^) were calculated according to [49]. Inflammatory conditions were applied with LPS by treating cells 2 h before exposure with 1 µg/mL. As a control for *IL8* induction 15 ng/mL TNF-α was applied to cells. Statistical information can be found in Supporting Information File 1 (Figure S7 and Figure S8).

Exposure of cells to 0.03 µg Ag/cm^2^ Ag NPs did not alter the expression of *TNFA* and *IL8*. However, 4 h after exposure elevated levels of *TNFA* expression were measured for cells exposed to 0.3 µg Ag/cm^2^ with a 14 ± 7 fold increase in expression. Furthermore, after 4 h *IL8* expression increased to 21 ± 10 fold for cells exposed to 0.3 µg Ag/cm^2^. However, both markers did not show an increased level of expression after 24 h anymore. As observed with ELISA, upon LPS treatment *TNFA* expression increased as well as *IL8*. Comparably, a synergistic effect could not be observed as Ag NP exposure to LPS-treated cells did not lead to a significantly different expression of the marker genes after 4 and 24 h. Cells treated with TNF-α showed a moderate increase of *IL8* as well as an induction of *TNFA* expression 4 h after treatment. After 24 h, a strong variation in *IL8* expression was found and expression of *TNFA* was not induced anymore, when cells were treated with TNF-α.

Oxidative stress, i.e., the expression of *SOD1* and *HMOX1,* was not altered 4 and 24 h after exposure. Moreover, when cells were treated with LPS, an expression of *SOD1* and *HMOX1* was not induced.

## Discussion

There is an urgent need for realistic exposure scenarios for in vitro toxicity testing of nanomaterials. We considered in this study the three main important points for inhalation risk assessment studies: realistic NP application simulating inhalation, NP concentration simulating occupational exposure, and a 3D in vitro lung-cell culture model simulating the human epithelial alveolar/airway barrier. In addition, the effects of aerosolized Ag NPs with Ag NPs applied in suspension were compared and by modelling the deposited Ag NP dose with the ISDD model it was possible to compare dose effects in two distinct exposure scenarios.

Currently three different strategies are used to investigate possible effects of inhaled Ag NPs. Most commonly Ag NPs are applied in dispersion onto different lung cells in vitro [22,30,37,50–51], animal models (mainly rodents) are used for inhalation [35,52–54] or instillation [55–56] of Ag NPs, and most recently also ALI exposures of lung cell cultures have emerged [41,57]. Due to the complexity of aerosol generation, measuring on-line the deposited NP dose and more sophisticated co-culture systems, ALI exposures are more complex to perform than the more simple suspension experiments. However, it allows for a controlled in vitro application of NPs in their native state as a defined aerosol directly onto cells and therefore reflects, in addition, a more realistic scenario. Furthermore, ALI exposures are less expensive, easier and quicker to perform than animal studies. In previous studies we have employed ALI exposures to investigate the toxic potential of various materials such as diesel car and scooter exhaust gases [58–59], zinc oxide [41], cerium oxide [60], gold (Au) [42] and silver [44]. Furthermore, the triple cell co-culture system has been evaluated in terms of its functional relevance in vivo and also allows studies at the ALI [40,61].

Only a few studies investigated the effects of Ag NPs at the ALI [44,57,62]. We have used the same cell cultures and similar endpoints as described in our previous studies [42,44] to easily compare the effects of different materials such as gold and silver and furthermore to compare different sizes and coatings of Ag NPs. The previous results were obtained with 20 nm citrate-coated Ag NPs [44] and 15 nm citrate-coated Au NPs [42]. In the present study we used the ALICE system to nebulize well-characterized [45–46] PVP-capped 100 nm Ag NPs. The majority of the 100 nm PVP-capped Ag NPs were found as aggregates inside vesicles, a finding which was similar for the 20 nm Ag NPs [44]. The aggregation was not as prominent for 15 nm Au NPs [42], and since similar concentrations of both materials were used a material-dependent aggregation can be assumed. No alteration of the cell morphology was observed in all studies. Furthermore, most of the NP exposures (i.e., independent of material, size, coating and concentration) were found to cause no cytotoxicity, nor could an alteration of cytokine release and oxidative stress marker expression be detected. In the current study, it has been observed, however, that an exposure to 0.3 µg Ag/cm^2^ 100 nm PVP-coated Ag NPs lead to an increase of cytokine expression. An increase could only be observed after 4 h. As a release of cytokines could not be detected with ELISA we assume that this is a transient and acute effect, which decreases to normal levels within a short time.

Dependent on how NPs are applied, i.e., either by submerged or ALI conditions, different toxicological results can be obtained [60]. Thus when comparing ALI with submerged exposures of 100 nm PVP-coated Ag NPs a number of differences were found. Cytotoxicity was observed for cells under submerged conditions at the two highest concentrations tested, in contrast to the ALI exposures. This outcome might be biased as LDH measurements can result in false positive or false negative results by high NP concentrations. Also for the other endpoints differences were observed for the suspension experiments after 24 h for the highest dose in the upper compartment as well as for the two lower doses in the lower compartment. The significant release of IL-8 is in contrast to any other studies we performed with all NPs at the ALI (i.e., Au NPs and also Ag NPs of different sizes), as an immune response could never be monitored with ELISA.

To compare the different approach of submerged vs ALI exposures it is important to relate any observed effect to the effectively deposited Ag NP concentration on the cells. The dose that was calculated with the ISDD model [47,63] for the suspension conditions reflects the NP concentration that the cells encounter. After 4 h at least 24% and after 24 h at least 71% of the total applied NP dose was deposited, which resulted in 0.6, 1.1 and 1.7 µg Ag/cm^2^ after 4 h, and 1.7, 3.4, and 5.1 µg Ag/cm^2^ after 24 h, respectively. Thus, 30 µg Ag/mL applied as dispersed solution exceeds the highest ALI concentration (3 µg Ag/cm^2^) by almost 70%. As Gangwal et al. recently reported in their ToxCast testing review based on occupational exposure potential [13], a realistic lung dose deposition of 5–100 nm Ag NPs after 24 h in a high environmental concentration scenario of 1 mg Ag/m^3^ ranges within 0.061–0.15 µg Ag/cm^2^. Furthermore, the calculated deposition for a full working lifetime exposure at a more realistic environmental concentration of 0.1 mg Ag/m^3^ was calculated to be in the range of 2.0–4.9 µg Ag/cm^2^ for Ag NPs with a diameter of 5–100 nm. Thus, the submerged exposures (i.e., the highest concentration after 4 h and all concentrations after 24 h), as well as the highest dose at the ALI (3 µg Ag/cm^2^) rather reflect a total deposited dose of a working lifetime exposure or even higher concentrations. As these quantities extend an acute exposure scenario concentration, realistic exposure conditions cannot be considered anymore, since short-term cytotoxic and inflammatory effects were analysed in this study. Other endpoints or in vivo models should be chosen for repeated low-dose administrations to address chronic and accumulative effects. A realistic exposure concentration with regard to the endpoints analysed is necessary and only this allows for a profound discussion of the realistic toxic potential of the materials applied. The dose exposed by submerged conditions would thus result in a calculated aerosol concentration 200 times higher than the threshold limit value of 0.1 mg Ag/m^3^ for Ag aerosols as set by the American Conference of Governmental Industrial Hygienists (ACGIH) [14]. A further aspect might be that the release pattern of pro-inflammatory proteins is different for ALI compared to submerged cell cultures. The released proteins might only slowly diffuse into the basal cell culture compartment and thus cannot spread easily as under submerged conditions, which would lead to a lower effect being observed.

When comparing submerged vs ALI exposures over the same concentration range, it is reasonable that different NP exposures result in different observed effects. The gradual deposition under submerged conditions combined with a possible ion effect in solution could explain the differences. Others found dissolution and thus a subsequent release of Ag ions from PVP-coated Ag NPs that were synthesized similar to the particles used in the current study [64]. Other studies using the same PVP-capped Ag NPs as we used, showed a similar aggregation pattern as we found [46]. Furthermore, cell proliferation and migration (chemotaxis) both decreased, and the release of cytokines was affected. Increased IL-8 and decreased IL-6 and vascular endothelial growth factor (VEGF) levels were detected at high Ag NP concentrations [65]. These studies however, were obtained with human mesenchymal stem cells (hMSCs) after treatment up to 7 d in higher concentrations than those we used here. It also cannot be ruled out that hMSCs are more sensitive to Ag NPs or the observed effects are due to the higher particle concentrations.

We have shown in an earlier study, in which cells were exposed to high concentrations of AgNO_3_ (equal to 3 µg Ag/cm^2^) at the ALI, that cytotoxic effects were observed after 4 h and diminished after 24 h [44], compared to 100 nm PVP-coated Ag NP submerged exposures, where an increased LDH release was measured 24 h after exposure. The short-term effects of Ag ions could be explained as Ag NPs are gradually releasing Ag ions in solution and therefore effects are only visible after a certain lag time, whereas an exposure to AgNO_3_ has an immediate effect on the cells. Moreover, aggravating effects under inflammatory conditions were only observed for high AgNO_3_ exposures. These results suggest a higher cytotoxic potential for Ag ions compared to Ag NPs as well as a gradual release of Ag ions might interfere with the biological environment over time. However, the estimation of the dissolution of Ag NPs under release of Ag^+^ in complex biological media is challenging. This is due to many possible interactions with the inorganic and organic components, e.g., precipitation as AgCl or complexation with proteins. Experimentally, it is not possible to measure the amount of free Ag^+^ in such systems. However, by using a simplified system, which contains major components of biological media, it was able to show that the dissolution in chloride-containing media is reduced, probably due to the formation of AgCl. In contrast, glucose has no significant effect. Cysteine as a sulphur-containing molecule can decelerate the dissolution process, possibly by blocking of the NP surface. Therefore, it can be assumed that the amount of free silver ions (i.e., neither precipitated nor complexed by proteins) from silver nanoparticles in biological media is small, in any case smaller than during dissolution in pure water [66].

## Conclusion

The exposure of Ag NPs at the air–liquid interface reflects a more realistic scenario for in vitro studies than addition of NPs in suspension. Our results indicated a significant difference between the two exposure methods with submerged cultures showing a stronger effect than ALI exposed cells and, thus, revealed the importance of an adequate experimental setup. In accordance, to study the effects of NPs in a biological environment, a complex cell culture model is necessary in order to reflect in vitro the versatile conditions in vivo. Furthermore, the limitations of in vitro exposures need to be taken into account when elucidating the effects of Ag NPs in lung cell cultures. The concentrations applied after 24 h under submerged conditions rather reflect a working lifetime than an acute exposure scenario and have shown a cytotoxic and pro-inflammatory effect. However, when analysing the effects in cell cultures only acute endpoints can be chosen, thus, interpretation of the data needs to be achieved in this regard. Chronic inhalation scenarios need prolonged low-dose applications. Regarding the general view of the performed experiments, no acute cytotoxicity and pro-inflammation activity of Ag NPs can be expected under realistic concentration scenarios, revealing a low impact of Ag NPs on human health. Nevertheless, secondary effects of Ag NPs, when incorporated in the biological environment over time, cannot be ruled out by in vitro experiments and chronic inhalation studies of Ag NPs need to be considered in the future.

## Experimental

### Cell culture

Experiments were carried out with a triple cell co-culture model of the human epithelial alveolar/airway barrier as described in detail by [40,61,67]. Briefly, A549 cells (adenocarcinomic human derived alveolar type II epithelial cells) were cultivated in Roswell Park Memorial Institute (RPMI) 1640 medium (w/25 mM HEPES, w/o L-Glutamine, Gibco, Life Technologies Europe B.V., Zug, Switzerland), supplemented with 1% penicillin G/streptomycin sulfate (P/S; 10,000 units/mL / 10,000 µg/mL, Gibco), 1% L-Glutamine (L-Glut; Life Technologies Europe) and 10% foetal bovine serum (FBS; PAA Laboratories, Chemie Brunschwig AG, Basel, Switzerland), subsequently referred to as “RPMI complete”. For exposure experiments, cells were seeded in BD Falcon™ cell culture inserts (high pore density PET membranes with a growth area of 4.2 cm^2^ and 3.0 µm pores in diameter; Becton Dickinson AG, Allschwil, Switzerland) placed in BD Falcon™ 6-well tissue culture plates at a density of 0.5 × 10^6^ cells/mL per insert. Cells were grown to confluence for 5 d under submerged conditions (2 mL RPMI complete in the upper and 3 mL in the lower transwell chamber). Peripheral blood monocytes were isolated from buffy coats (Blood donation service SRK Bern AG, Switzerland) using Lymphoprep™ density gradients and CD14+ MicroBeads (Miltenyi Biotec GmbH, Bergisch Gladbach, Germany) according to the manufacturer's manual. For the generation of monocyte-derived dendritic cells (MDDCs), the monocytes were cultured for 7 d in RPMI complete with additional supplementation of 10 ng/mL IL-4 (R&D Systems Europe Ltd., Abingdon, UK) and 10 ng/mL GM-CSF (R&D Systems). Monocyte-derived macrophages (MDMs) were obtained by culturing the monocytes for 7 d in RPMI complete containing 10 ng/mL M-CSF (R&D Systems).

The triple cell co-cultures were set together as described in detail [68]. 2.5 × 10^5^ MDDCs at the basal side and 5 × 10^4^ MDMs at the apical side of the insert were added. After cultivation for 24 h in the incubator the cells were used for submerged exposures or transferred to ALI conditions. The cell culture medium from the upper transwell chamber was removed and the cell culture medium in the lower transwell chamber was replaced with 1.2 mL RPMI complete. Cells were exposed after an additional 24 h in the incubator at the ALI. In some of the experiments, an inflammatory environment was created by adding 1 µg/mL lipopolysaccharide (LPS) (Pseudomonas aeruginosa, Sigma-Aldrich Chemie GmbH, Buchs, Switzerland) into the medium of the lower transwell chamber 2 h before exposure [42,44].

### Exposure conditions

Cells were exposed under submerged conditions by applying in the upper transwell chamber 1 mL of the appropriate NP suspension in RPMI complete freshly prepared before the experiment. The media on the lower transwell chamber was exchanged as well with 2 mL fresh RPMI. Cells were exposed at the ALI using the air–liquid interface cell exposure system (ALICE) as previously described in [41–42,44]. A volume of 1 mL of the appropriate Ag NP suspension was nebulized onto the cells. Subsequently, the cells were kept either under submerged conditions or at the ALI for post-exposure incubation times of 4 and 24 h in 5% CO_2_ humidified atmosphere at 37 °C. Further end-point analysis of supernatants was conducted with basal (ALI and submersed) and apical (submersed) cell culture media.

#### Silver nanoparticles

PVP-coated Ag NPs were synthesized by reduction with glucose in the presence of PVP according to Wang et al. [69] and have been used already before [45–46]. Briefly, 2 g of glucose and 1 g of PVP were dissolved in 40 g of water and heated to 90 °C. Then, 0.5 g of AgNO_3_ dissolved in 1 mL of water was quickly added. The dispersion was kept at 90 °C for 1 h and then cooled to room temperature. The particles were collected by ultracentrifugation (30,000 rpm; 30 min), redispersed in pure water and collected again by ultracentrifugation. Thereby, NO_3_^−^, excess glucose and its oxidation products, excess PVP, and excess Ag^+^ were fully removed. The silver nanoparticles were then redispersed in water. The final silver concentration was determined by atomic absorption spectroscopy (AAS). Polyvinylpyrrolidone (PVP K30, Povidon 30; Fluka, molecular weight 40,000 g/mol), silver nitrate (Roth, p.a.), and D-(+)-glucose (Sigma-Aldrich) were used. Ultrapure water was prepared with an ELGA Purelab ultra instrument. Suspensions for exposure were adjusted to 24, 240 and 2400 µg Ag/mL by dilution in double-distilled H_2_O or via ultrafiltration by using 30 kDa MWCO centrifugal filter units (Vivaspin 20; Sartorius Stedim AG, Tagelswangen, Switzerland) at 3000*g* by diafiltration for 10 min. The Ag NP dispersions were always freshly prepared before each individual experiment.

#### Nanoparticle characterisation

The size distribution and zeta potential of the Ag NP stock solutions (in water) were analysed by dynamic light scattering using a Malvern Zetasizer Nano ZS. The polydispersity index (PDI) was below 0.3 in all cases, indicating a good dispersion of the particles and only little agglomeration. The concentration of silver was determined by atomic absorption spectroscopy (AAS; Thermo Electron Corporation, M-Series). The detection limit was 1 μg/L. Scanning electron microscopy (SEM) was performed with a FEI Quanta 400 ESEM instrument in high vacuum without sputtering on silicon sample holders. As demonstrated earlier, these Ag NPs are well dispersed in protein containing cell culture medium (RPMI + 10% FCS) [70].

#### Real-time reverse-transcriptase polymerase chain reaction (real-time RT-PCR)

As described in [44], following post-incubation of Ag NP and AgNO_3_ exposures for 4 h and 24 h, the insert membranes were cut out, transferred immediately into RNAprotect cell reagent (Qiagen AG, Hombrechtikon, Switzerland) and stored at 4 °C until further processing. Cells were detached by vortexing and lysed by centrifuging with QIAshredder columns (Qiagen). Total RNA was isolated by using the RNeasy plus kit (Qiagen) according to the manufacturer’s guidelines and the RNA concentration was determined by a NanoDrop 2000 (Thermo Scientific, Witec AG, Littau, Switzerland). Reverse transcription (incubation 1 h at 37 °C) was carried out with the Omniscript Reverse Transcription Kit (Qiagen) in 10 µL volume with 0.25 µg RNA/reaction, by using a master mix consisting of 0.25 mM of each dNTP (Qiagen), 0.5 µM Oligo-dT primers (Qiagen), 10 units RNase inhibitor (RNasin Plus RNase Inhibitor, Promega AG, Dübendorf, Switzerland), 2 units Omniscript Reverse Transcriptase (Qiagen) and 1× buffer RT (Qiagen). Real-time PCR was performed in a reaction volume of 10 µL, with a total of 2 µL of tenfold diluted cDNA, by using a Fast SYBR Green master mix (Applied Biosystems, Life Technologies Europe B.V., Zug, Switzerland) with a 50 nM primer mix in a 7500 Fast real-time PCR system (Applied Biosystems). Settings: Denature 20 s at 95 °C, PCR cycles (40): 3 s at 95 °C, 30 s at 60 °C. Relative expression levels were calculated by using the ^ΔΔ^Ct method as described elsewhere [49] with glyceraldehyde 3-phosphate dehydrogenase (*GAPDH*) [GenBank: NC_000012] as internal reference gene. The expression levels of heme-oxygenase 1 (*HMOX1*) [GenBank: CP002685], superoxide dismutase 1 (*SOD1*) [GenBank: NM_000454], interleukin-8 (*IL8*) [GenBank: NM_000584] and tumor necrosis factor alpha (*TNFA*) [GenBank: NM_000594] were determined. Primer sequences (Microsynth AG, Balgach, Switzerland) were the following: *GAPDH*: forward 5’- AAC AGC CTC AAG ATC ATC AGC-3’, reverse 5’- GGA TGA TGT TCT GGA GAG CC-3’; *HMOX1*: forward 5’- TTC TCC GAT GGG TCC TTA CAC T-3’, reverse 5’- GGC ATA AAG CCC TAC AGC AAC T-3’; *SOD1*: forward 5’- GTG CAG GTC CTC ACT TTA AT-3’, reverse 5’- CTT TGT CAG CAG TCA CAT TG-3’; *IL8*: forward 5’- CTG GCC GTG GCT CTC TTG-3’, reverse 5’- CCT TGG CAA AAC TGC ACC TT-3’; *TNFA*: forward 5’- CCC AGG GAC CTC TCT CTA ATC A-3’, reverse 5’- GCT ACA GGC TTG TCA CTC GG-3’.

#### Lactate dehydrogenase release

As a general measure for cytotoxicity, the release of lactate dehydrogenase (LDH) was assessed as described in [44]. For that, the medium of the lower transwell (ALICE) and from the upper as well as the lower transwell (suspension exposures) were collected 4 h and 24 h after exposure and stored at 4 °C for analysis. The LDH cytotoxicity detection kit (Roche Applied Science, Mannheim, Germany) was used according to the supplier’s manual. LDH was quantified photometrically by measuring at 490 nm, with 630 nm as reference wavelength. Each sample was assessed in triplicate. The values were expressed as fold increase related to the incubator control at appropriate post-exposure times. For positive controls co-cultures were exposed to 0.2% Triton X-100 detergent in H_2_O at 37 °C for the same duration as samples were post-incubated.

#### Chemokine/cytokine quantification

The released pro-inflammatory proteins IL-8 and TNF-α were quantified with a commercially available DuoSet ELISA Development Kit (R&D Systems) according to the manufacturer’s protocol. LPS (1 μg/mL) and TNF-α (15 ng/mL; Sigma-Aldrich) served as positive control to induce the release of TNF-α and IL-8, respectively.

#### Laser scanning microscopy

As described in [44], the triple cell co-cultures were fixed on the cell culture insert with 3% paraformaldehyde in phosphate buffered saline (PBS) for 15 min at room temperature and then treated with 0.1 M glycine in PBS for 10 min. Before staining, the cells were permeabilised with 0.2% Triton X-100 in PBS for 15 min at room temperature. The cytoskeleton (i.e., F-actin-filaments of all cells) was stained with rhodamine phalloidin 1:100 (R-415; Molecular Probes, Life Technologies Europe B.V., Zug, Switzerland) and DNA was stained with DAPI 1 μg/mL (Sigma-Aldrich). Preparations for optical analysis were mounted in Glycergel (DAKO Schweiz AG, Baar, Switzerland). The samples were visualized with an inverted Zeiss laser scanning microscope (LSM) 710 (Axio Observer.Z1, Lasers: HeNe 633 nm, and Ar 488 nm). Minimum intensity projections of z-stacks of phase contrast images were processed with Fiji. Samples with no fluorescence labelling were used to adjust the background parameters for the stained cells in order to avoid unspecific signals from the Ag NPs. Images are processed with Adobe Photoshop and Adobe Illustrator.

#### Transmission electron microscopy

As described in [44], intracellular particles were visualized by conventional TEM. For TEM analysis, the exposed cells on the transwell membrane were fixed with 2.5% glutaraldehyde in 0.03 M potassium phosphate buffer for at least 24 h, subsequently washed with potassium phosphate buffer and post-fixed with 1% osmium tetroxide in sodium cacodylate buffer, washed with maleate buffer, and stained en bloc with 0.5% uranyl acetate in maleate buffer. Afterwards, the cells were dehydrated in ascending ethanol series, and embedded in epon [71]. From the embedded cells, ultrathin sections were cut parallel to the vertical axis of the inserts, mounted on copper grids and stained with uranyl acetate. Imaging was done with a Philips CM12 TEM (FEI Co Philips Electron Optics).

#### Estimation of NP dosage in dispersion over time

The in vitro sedimentation, diffusion and dosimetry model (ISDD) according to Hinderliter et al. was used [47] to estimate the effective Ag NP dose delivered to the cells. This model estimates the NP dose delivered to cells as a function of time, by using parameters such as the size, density and aggregation state of the NPs as well as the temperature and height of the cell-culture medium. The following parameters were used as input for the ISDD model: particle hydrodynamic diameter = 116 nm, particle core size = 60 nm, effective mass density = 7.77 g/cm^3^, media height = 2.38 mm, temperature = 37 °C, medium density = 1.00 g/mL, and viscosity = 0.00074 Pa s.

#### Statistics

All data are presented as the mean ± standard error of the mean (SEM). Statistical analysis was performed with GraphPad Prism 5 (GraphPad Software Inc., La Jolla, California, USA). A two-way analysis of variance (ANOVA) with a subsequent Dunnett’s post-hoc test was performed. Values were considered significantly different with *p* < 0.05 (*), *p* < 0.01 (**).

## Supporting Information

Supporting Information features additional statistical, such as numerical values and confidence intervals.

File 1 Statistical information.
