# Supplementary material for: Mimicking exposures to acute and lifetime concentrations of inhaled silver nanoparticles by two different in vitro approaches
Source: Beilstein J Nanotechnol. 2014 Aug 26;5:1357–70. doi: 10.3762/bjnano.5.149 (PMC4168965; doi:10.3762/bjnano.5.149)
Supplement: File 1 — Statistical information. [file Beilstein_J_Nanotechnol-05-1357-s001.pdf]

Supporting Information

for

Mimicking exposures to acute and lifetime concentrations of inhaled silver nanoparticles by two different in vitro approaches

Fabian Herzog[1], Kateryna Loza[2], Sandor Balog[1] Martin J. D. Clift[1], Matthias Epple[2], Peter Gehr[3], Alke Petri-Fink[1,4] and Barbara Rothen-Rutishauser*[1,5]

Address: [1]Adolphe Merkle Institute, BioNanomaterials, University of Fribourg, Rte de l'Ancienne Papeterie, CP 209, 1723 Marly, Switzerland, [2]Inorganic Chemistry and Center for Nanointegration Duisburg-Essen (CeNIDE), University of Duisburg-Essen, Universitaetsstrasse 5-7, 45141 Essen, Germany, [3]Institute of Anatomy, University of Bern, Baltzerstrasse 2, 3012 Bern, Switzerland, [4]Department of Chemistry, University of Fribourg, Chemin du Musée 9, 1700 Fribourg, Switzerland and [5]Respiratory Medicine, Department of Clinical Research, Inselspital University Hospital, University of Bern, Murtenstrasse 50, 3008 Bern, Switzerland

Email: Fabian Herzog - fabian.herzog@unifr.ch; Kateryna Loza - kateryna.loza@uni-due.de; Sandor Balog - sandor.balog@unifr.ch; Martin J. D. Clift - martin.clift@unifr.ch; Matthias Epple - matthias.epple@uni-due.de; Peter Gehr - peter.gehr@ana.unibe.ch; Alke Petri-Fink - alke.fink@unifr.ch; Barbara Rothen-Rutishauser* - barbara.rothen@unifr.ch

* Corresponding author

Statistical information

LDH statistical information

Air-liquid

	4h			24h		
	Mean	SEM	N	Mean	SEM	N
0 LPS	1.183208	0.2300672	7	1.12395	0.1868752	7
0 Triton	12.03719	2.384554	7	6.369139	2.858138	6
0.03	1.59628	0.2099543	7	1.174683	0.1299934	7
0.03 LPS	1.209883	0.2159392	7	1.005751	0.07397007	7
0.3	2.029889	0.544067	7	1.187982	0.1149638	7
0.3 LPS	1.508786	0.3255128	7	1.133313	0.1525453	7
3	1.514661	0.2611198	4	2.701968	1.281242	4
3 LPS	2.23911	1.152152	4	1.598825	0.3680741	4
0 TNF- α	1.056038	0.2848305	6	1.295242	0.2425573	6

Submerged apical

	4h			24h		
	Mean	SEM	N	Mean	SEM	N
0 LPS	0.9376091	0.1551273	4	0.9633195	0.1190512	4
0 Triton	8.961942	1.672538	4	7.621901	2.472347	4
10	1.229932	0.2359734	4	1.650983	0.07809889	4
10 LPS	1.248155	0.1622663	4	1.547896	0.1388992	4
20	1.127318	0.2636101	4	2.015599	0.5261061	4
20 LPS	0.9905616	0.2143921	4	2.163305	0.6185693	4
30	0.9446509	0.1087741	4	1.903531	0.304055	4
30 LPS	0.9715688	0.1600173	4	1.911844	0.5342641	4
0 TNF- α	0.9539278	0.2191581	4	1.465547	0.2630565	4

Submerged basal

	4h			24h		
	Mean	SEM	N	Mean	SEM	N
0 LPS	0.9748341	0.02070217	4	0.8898862	0.05228264	4
0 Triton	8.973631	1.719703	4	6.850787	1.394784	4
10	1.024721	0.2008674	4	1.080621	0.1215216	4
10 LPS	1.078626	0.1722315	4	1.300669	0.2915927	4
20	1.172452	0.09105235	4	1.318276	0.2725257	4
20 LPS	1.0343	0.08879732	4	1.232829	0.3723384	4
30	1.037678	0.14869	4	1.59003	0.1183439	4
30 LPS	1.119406	0.1143621	4	1.391683	0.349377	4
0 TNF- α	0.9271843	0.08901137	4	1.218904	0.1991518	4

Figure S1: LDH numerical values (Figure 4).

Air-liquid

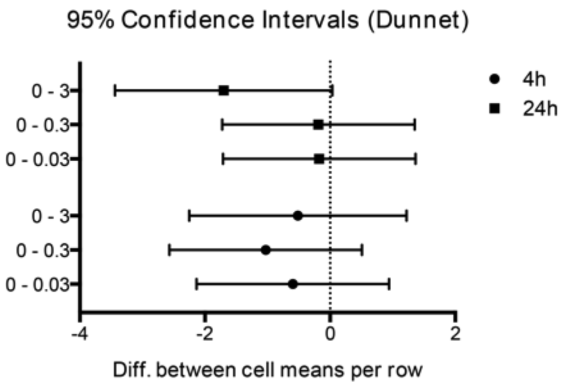

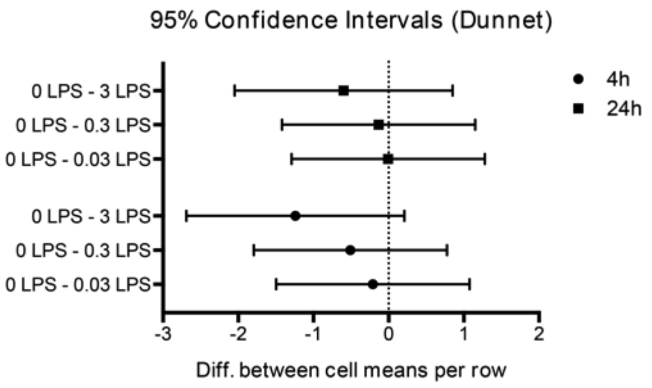

Submerged apical

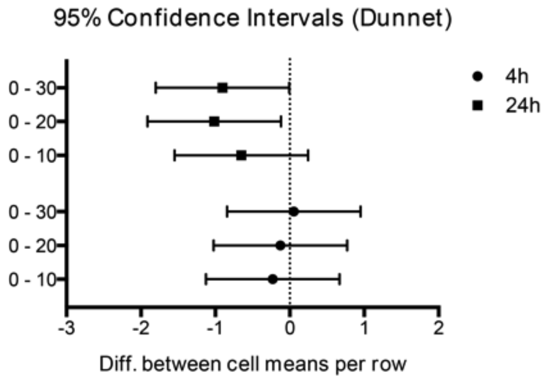

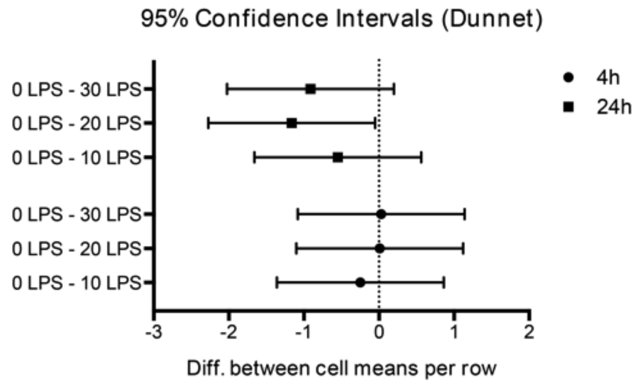

Submerged basal

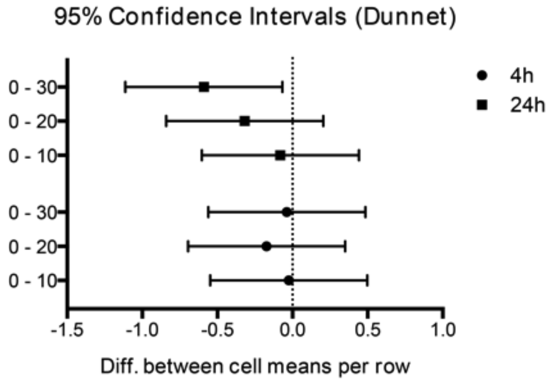

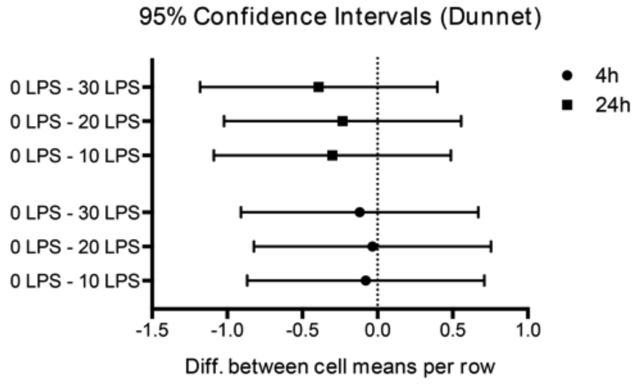

Figure S2: LDH confidence intervals (Figure 4).

ELISA statistical information

Air-liquid

	4h			24h		
	Mean	SEM	N	Mean	SEM	N
0	0.002396949	0.001191583	7	0.005954469	0.002047371	7
0.03	0.002757143	0.001690993	7	0.008109765	0.002820223	7
0.3	0.004042857	0.002433678	7	0.006398785	0.00211618	7
3	0.007444272	0.001597416	4	0.022649	0.01795702	4
0 LPS	1.14761	0.2682931	7	0.5867763	0.174216	7
0.03 LPS	0.8200057	0.1628574	7	0.3790922	0.06138428	7
0.3 LPS	0.7556536	0.157138	7	0.4310234	0.1015208	7
3 LPS	0.8707404	0.1893692	4	0.4428761	0.1835357	4

Submerged apical

	4h			24h		
	Mean	SEM	N	Mean	SEM	N
0	0.0072625	0.002152845	4	0.006275	0.003306341	4
10	0.10435	0.09592507	4	0.0197525	0.01178621	4
20	0.1176575	0.09962424	4	0.06679	0.04416129	4
30	0.0610725	0.02907832	4	0.0129425	0.00294584	4
0 LPS	0.8929375	0.1519856	4	0.684915	0.2521508	4
10 LPS	0.88796	0.181741	4	0.785885	0.2761415	4
20 LPS	0.829955	0.2192534	4	0.737545	0.2638289	4
30 LPS	1.035245	0.205232	4	0.6993925	0.2216794	4

Submerged basal

	4h			24h		
	Mean	SEM	N	Mean	SEM	N
0	0.008933083	0.004664426	4	0.005943096	0.002366219	4
10	0.02038375	0.01147082	4	0.01993881	0.01255014	4
20	0.1335115	0.1210377	4	0.1062834	0.08153138	4
30	0.3865929	0.3769997	4	0.03284117	0.01210865	4
0 LPS	1.608674	0.5312613	4	1.647491	0.6474342	4
10 LPS	0.9355566	0.4701209	4	1.838979	0.558663	4
20 LPS	1.405756	0.5893943	4	1.629033	0.6058467	4
30 LPS	1.804887	0.5562839	4	1.551325	0.4807593	4

Figure S3: TNF-α ELISA numerical values (Figure 5).

Air-liquid

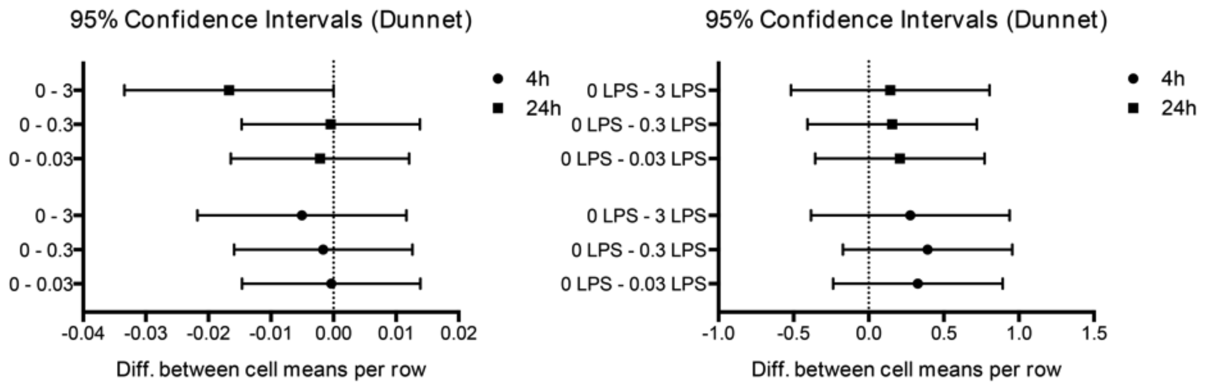

Submerged apical

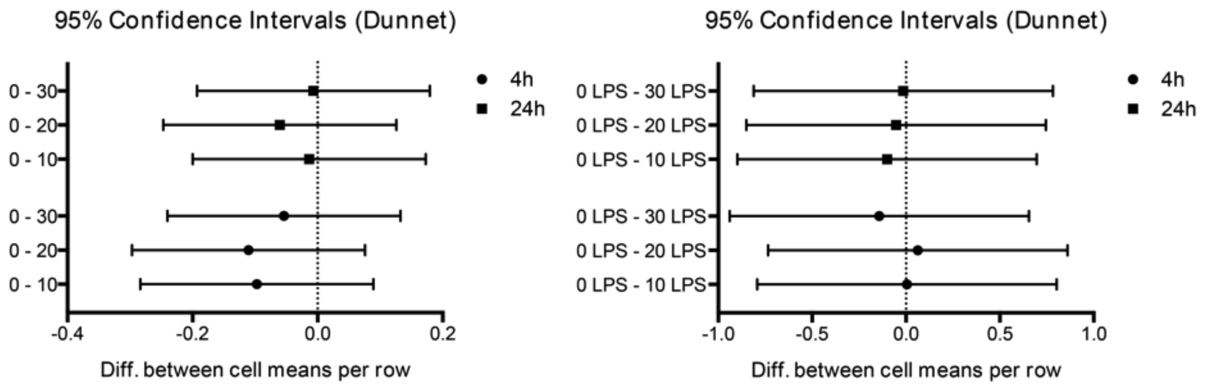

Submerged basal

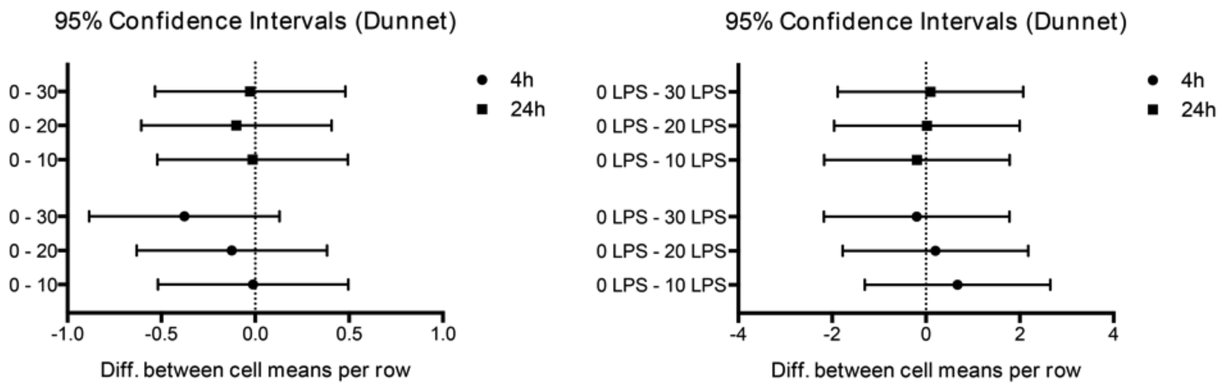

Figure S4: TNF-α ELISA confidence intervals (Figure 5).

Air-liquid

	4h			24h		
	Mean	SEM	N	Mean	SEM	N
0	1.549731	0.5286818	6	11.22107	2.914826	6
0.03	2.546267	0.6463377	6	12.55854	2.399343	6
0.3	3.827393	0.6624322	6	16.28226	3.149896	6
3	5.015773	1.136081	4	18.29991	3.408101	4
0 LPS	24.4936	4.087953	6	40.2624	5.239343	6
0.03 LPS	26.2643	4.590001	6	40.52784	5.325487	6
0.3 LPS	30.02083	5.657012	6	40.26498	5.714561	6
3 LPS	23.87286	5.861775	4	35.20732	7.170444	4
0 TNF- α	19.22579	4.693774	5	35.56175	6.415161	5

Submerged apical

	4h			24h		
	Mean	SEM	N	Mean	SEM	N
0	1.359866	0.510445	4	5.538367	2.361131	4
10	2.206811	0.6354272	4	9.921396	3.480271	4
20	2.292276	0.64626	4	10.83645	2.318764	4
30	2.184406	0.6165758	4	15.33042	3.71372	4
0 LPS	9.533554	1.699914	4	15.67188	2.968246	4
10 LPS	8.394997	1.808815	4	14.92238	3.265774	4
20 LPS	7.854643	0.7053187	4	16.0126	4.157615	4
30 LPS	8.011962	0.9322535	4	9.236403	3.619916	4
0 TNF- α	8.485684	3.393147	4	14.34096	3.408254	4

Submerged basal

	4h			24h		
	Mean	SEM	N	Mean	SEM	N
0	0.9961734	0.177912	4	6.458008	2.182904	4
10	1.54031	0.499004	4	13.97257	4.130198	4
20	2.284887	1.161869	4	14.37367	1.105503	4
30	2.453401	1.232491	4	12.04253	2.516031	4
0 LPS	9.793168	3.755139	4	27.15633	3.423011	4
10 LPS	7.492945	2.962165	4	28.11841	4.925918	4
20 LPS	8.738611	3.699433	4	26.72217	4.865003	4
30 LPS	9.399978	3.434271	4	27.9281	4.984178	4
0 TNF- α	5.886376	1.835631	4	28.6316	6.301556	4

Figure S5: IL-8 ELISA numerical values (Figure 5).

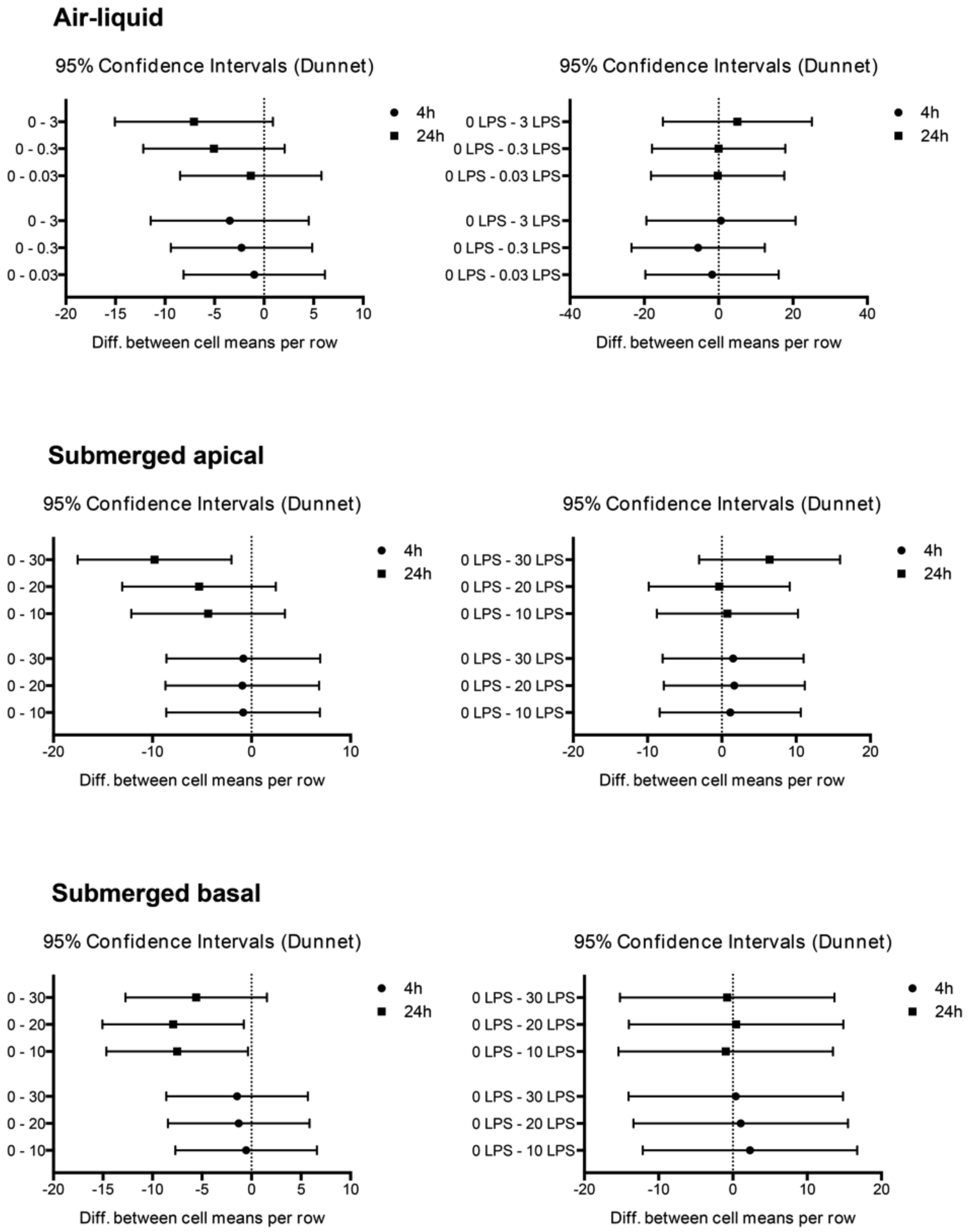

Figure S6: IL-8 ELISA confidence intervals (Figure 5).

Real-time RT-PCR statistical information

Air-liquid 4h

	TNF-α			IL-8			HMOX-1			SOD-1		
	Mean	SEM	N	Mean	SEM	N	Mean	SEM	N	Mean	SEM	N
0.03	3.380989	0.9192738	4	2.629355	0.6822194	4	1.214474	0.4239489	4	1.994697	1.294724	4
0.3	13.68572	6.859244	4	21.08179	9.979162	4	3.936073	2.877128	4	1.635696	0.8887775	4
0 LPS	13.02216	7.596604	4	40.54984	19.25233	4	1.225706	0.4650098	4	1.419569	0.1964079	4
0.03 LPS	13.04659	2.942452	4	49.07214	26.51158	4	0.4917439	0.08347811	4	1.460708	0.8934079	4
0.3 LPS	13.87112	4.837963	4	50.90971	17.9249	4	0.5910786	0.1238672	4	0.6180258	0.2575335	4
TNF-α	17.2677	15.89096	3	14.58669	8.748199	3	0.5660224	0.1465191	4	0.8871207	0.2933809	4

Air-liquid 24h

	TNF-α			IL-8			HMOX-1			SOD-1		
	Mean	SEM	N	Mean	SEM	N	Mean	SEM	N	Mean	SEM	N
0.03	2.382542	0.8100402	3	3.611365	3.029494	3	1.179974	0.2965065	3	1.092582	0.2239712	3
0.3	3.837958	2.211317	3	0.720722	0.5128186	3	1.464395	0.4357439	3	1.776737	0.6121695	3
0 LPS	11.34615	5.855048	4	15.6601	3.524914	4	1.009881	0.2115368	4	2.246957	0.7774521	4
0.03 LPS	6.238909	2.579321	3	15.92512	9.566056	3	1.522587	0.3370367	3	1.258192	0.3432497	3
0.3 LPS	6.463327	4.39348	4	19.29043	6.630352	3	0.8893991	0.2740776	4	1.049743	0.2276793	4
TNF-α	3.274363	1.316628	3	31.00285	28.28897	3	1.175377	0.2232322	4	1.83737	0.5806355	4

Figure S7: Real-time RT-PCR numerical values (Figure 6).

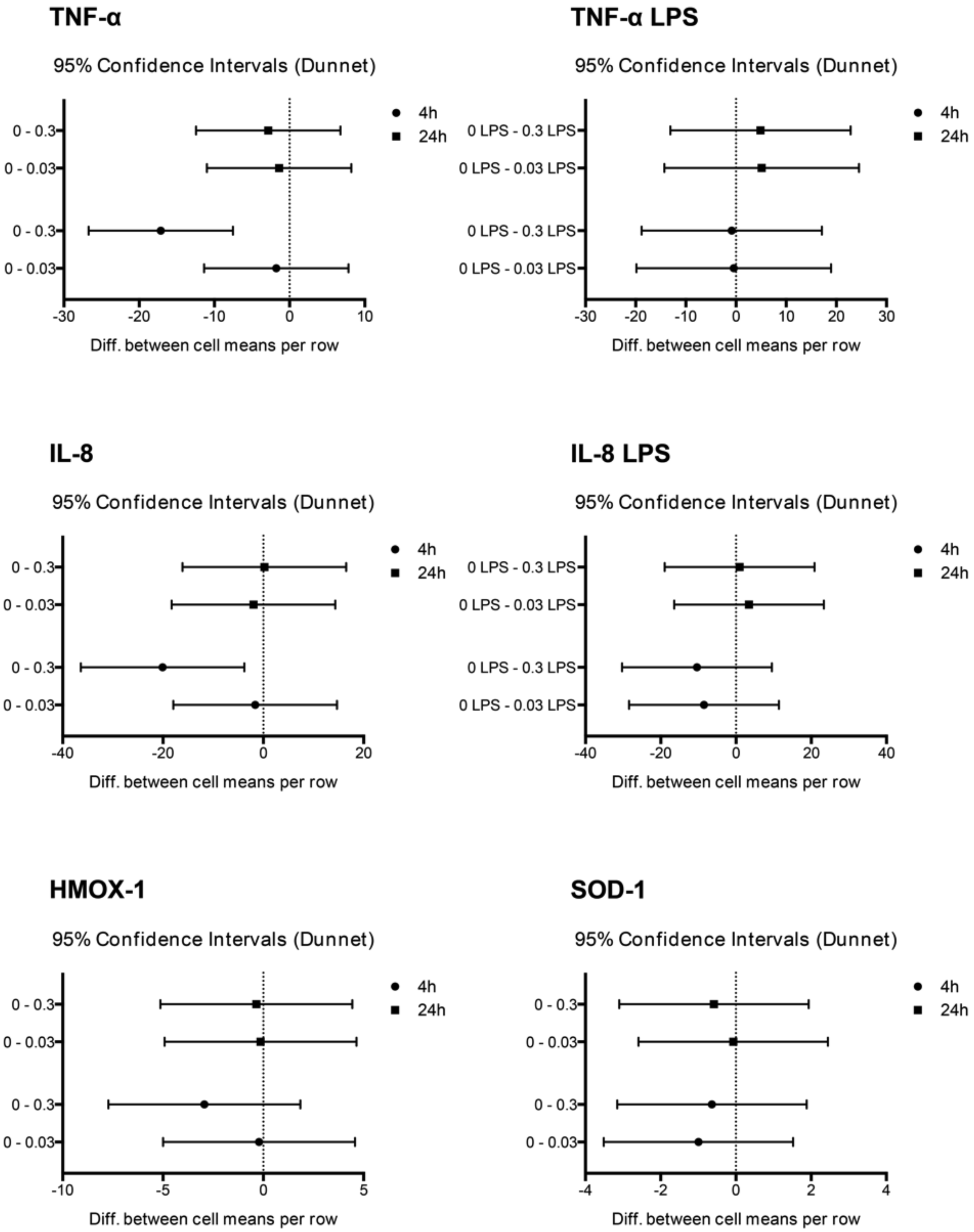

Figure S8: Real-time RT-PCR confidence intervals (Figure 6).
